# Characterization of Uterine Cervix Cancers in Women from Appalachian Kentucky

**DOI:** 10.3389/fonc.2021.808081

**Published:** 2021-12-09

**Authors:** Charles A. Kunos, Denise Fabian, Mahesh Kudrimoti, Rachel W. Miller, Frederick R. Ueland, Marcus E. Randall

**Affiliations:** ^1^ Department of Radiation Medicine, University of Kentucky, Lexington, KY, United States; ^2^ Department of Obstetrics and Gynecology, Division of Gynecologic Oncology, University of Kentucky, Lexington, KY, United States

**Keywords:** cervix cancer, radiotherapy, Appalachian Kentucky, brachytherapy, cervical cancer

## Abstract

Uterine cervix cancer (UCCx) is clinically and socioeconomically diverse among women in the United States (US), which obscures the discovery of effective radiochemotherapy approaches for this disease. UCCx afflicts 7.5 per 100,000 American women nationally but 11.7 per 100,000 women in Appalachian Kentucky (AppKY), when age-adjusted to the 2000 US standard population. Epidemiological chart review was performed on 212 women with UCCx treated at the University of Kentucky (UKY) between January 2001 and July 2021. Demographics, tumor characteristics, and relative radiochemotherapy dose and schedule intensity were compared among AppKY and non-AppKY cohorts as well as Surveillance, Epidemiology, and End Results (SEER) data. One hundred thirty-eight (65%) of 212 women seeking radiochemotherapy treatment for UCCx resided in AppKY. Most (80%) sought external-beam radiochemotherapy close to their AppKY residence. Brachytherapy was then most frequently (96%) conducted at UKY. Cancer stage at diagnosis was significantly more advanced in AppKY residents. Women residing in AppKY had a median 10-week radiochemotherapy course, longer than an 8-week guideline. Estimated survival in women residing in AppKY was 8% lower than US national averages. In summary, this study identified an increased percentage of advanced-stage UCCx cancer at diagnosis arising in AppKY residents, with a confounding population-specific delay in radiochemotherapy schedule intensity lowering survival.

## Introduction

Uterine cervix cancer (UCCx) ranks 20th (0.8%) among all new cancer cases in the United States (US), with 14,480 new cases estimated annually [7.5 per 100,000, age-adjusted to the 2014–2018 US standard population, ref. (1)]. The Commonwealth of Kentucky is highest among US states in UCCx incidence [9.6 per 100,000, age-adjusted to the 2000 US standard population, ref. (2)], and the Appalachian region of Kentucky (AppKY) drives this extraordinary health burden [11.7 per 100,000, age-adjusted, ref. (2)]. In the US, UCCx disproportionately afflicts women of minority groups or women of low socioeconomic status, partly as a consequence of insufficient access to and knowledge of UCCx screening programs (3).

Women with early-stage UCCx undergo radical surgery for cure, with adjuvant radiation indicated for large (2–4 cm) tumors, deep third stromal or parametrial invasion, and lymph node metastases (4–7). Bulky (≥4 cm) early-stage or advanced-stage (IIB-IVA) diseases at diagnosis are usually treated by external-beam radiotherapy plus platinum-based chemotherapy followed by intracavitary brachytherapy (8–13). Prior studies reported improved survival among women with advanced-stage UCCx who received a dose-schedule intensity of five or more weekly cycles of cisplatin (40 mg m^-2^) during an 8-week course of radiotherapy (12–14). US National Cancer Institute intervention trials study UCCx outcomes by accruing subjects nationwide, but such trials infrequently enroll residents in AppKY where new UCCx cases are extremely prevalent.

We undertook this retrospective study of UCCx among residents of AppKY to provide clinicopathological characterization of this disease in women from this region. This enabled us to test the hypothesis that UCCx clinicopathological factors in women from AppKY are distinct from the US general population, which may help explain the AppKY region’s extremely high UCCx incidence and mortality. For context, our data are in contrast to UCCx profiles found in the Surveillance, Epidemiology, and End Results database [SEER, ref. (1)].

## Materials and Methods

### Patient Cohorts

The UKY Institutional Review Board (Lexington, KY, protocol # 69443) approved this retrospective study. Cohorts consisted of a random sampling of women aged 18 years or older who were diagnosed with International Federation of Gynecology and Obstetrics (FIGO 1988) stages I to IVB UCCx treated by radiotherapy between January 2001 and July 2021 (*n* = 212 [25%] of 852 registered UKY UCCx patients). The FIGO 1988 UCCx cancer stage at diagnosis classification was uniformly applied for consistency over the 2001 to 2021 retrospective study period. UKY and its partnering community oncology practices serve the cancer clinical and research needs of an urban manufacturing and rural agricultural region settled by 4.5 million people in central and eastern Kentucky. For this study, only UCCx squamous, adenosquamous, and adenocarcinoma histologies were included. Women could not have had previous or synchronous invasive cancers. Women may have undergone total extrafascial hysterectomy and salpingo-oophorectomy with or without pelvic and para-aortic staging lymphadenectomy. However, to be included in this study, surgical patients also must have had indications for adjuvant radiotherapy such as a combination of positive angiolymphatic invasion, deep third stromal invasion, or large (2–4 cm) tumor size (5, 6), or, for adjuvant radiotherapy with chemotherapy, must have had bulky (>4 cm) tumor with parametrial tumor invasion, positive surgical margins, or lymph nodes with metastases (7). Women undergoing definitive intent external-beam radiotherapy with chemotherapy followed by brachytherapy were included whether they finished the intended course or not.

**Figure 1:**
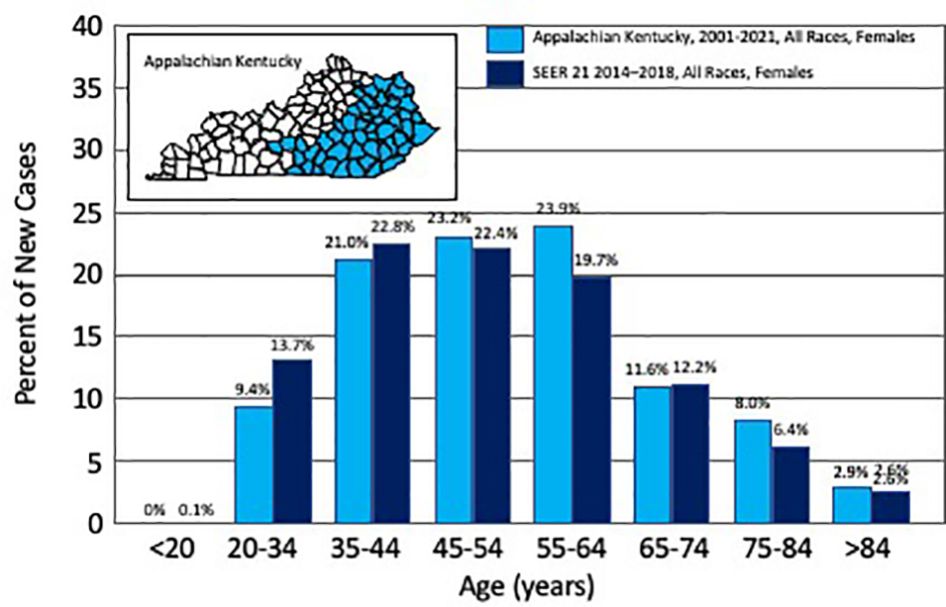
Percent of new uterine cervix cancer cases by age. Depicted are the number of new incident cases of uterine cervix cancer by age, as reported for Appalachian Kentucky (2001–2021) and for Surveillance, Epidemiology, and End Results (SEER 21, 2014–2018, ref. (1)). The inset identifies in blue the Appalachian counties in the Commonwealth of Kentucky.

Women were segregated into AppKY (generally, eastern Kentucky) or non-AppKY (generally, central Kentucky) cohorts by residence using various confirmatory identifiers, including address, zip code, or county of residence (**Figure 1** inset). UKY Markey Cancer Center provided all demographic and follow-up data for this study in a deidentified manner. The combined cohorts had a median follow-up of 29 months and 46% of patients were alive at the time of submission. Typical of our AppKY catchment, 98% were of Caucasian (white) race and 62% were current smokers.

### Treatment

Radiotherapy involved external-beam irradiation 5 days a week using either opposed anteroposterior or four-field portals (4,500 cGy) that in general spanned superoinferiorly the L4−L5 vertebral interspace to the lower third of the obturator foramina and laterally 2 cm beyond the pelvic brim, or intensity-modulated radiation therapy (IMRT). Low-dose-rate (LDR 8,000 cGy) or high-dose-rate (HDR 7,000 cGy) intracavitary or interstitial brachytherapy were prescribed to conventional point A or an individualized contoured UCCx tumor volume (**Table 1**). Radiotherapy variations were scored as minor, major but acceptable, or major and unacceptable, if the dose variation differed by less than 7%, 7% to 20%, or more than 20%, respectively, from the prescription dose or duration of treatment. Cisplatin (40 mg m^-2^) chemotherapy, not to exceed 70 mg total per week, was infused intravenously once a week during external-beam radiotherapy for six maximum cycles. Standardized total extrafascial hysterectomy with bilateral salpingo-oophorectomy might have been done for early-stage (IA-IIA) disease, or postradiotherapy as intended to reduce UCCx central pelvic relapse (IB-IIB). Surgical staging of lymph nodes was optional but recommended (**Table 1**).

**Table 1:** Demographics and tumor characteristics of patient population (*n* = 212).

Characteristic	Number (percent)*		
	AppKY (*n* = 138 [65])	Non-AppKY (*n* = 74 [35])	*p*-value
Mean age ± SE (range), years	54 ± 1 (23–88)	56 ± 2 (27–82)	0.41
Race			0.18
White	136 (99)	70 (95)	
Black or African American	2 (1)	4 (5)	
Ethnicity			0.02
Hispanic or Latino	3 (2)	8 (11)	
Non-Hispanic or Latino	135 (98)	66 (89)	
Religion			0.79
Christian	127 (92)	67 (91)	
Non-Christian	0 (0)	0 (0)	
Unaffiliated	11 (8)	7 (9)	
Smoking Habit			0.89
Current	89 (64)	42 (57)	
Cell Type			0.38
Squamous	95 (69)	44 (59)	
Adenosquamous	9 (6)	7 (10)	
Adenocarcinoma	34 (25)	23 (31)	
Stage at Diagnosis (FIGO 1988)			0.12
IA	2 (1)	3 (4)	
IB1	8(6)	6 (8)	
IB2	32 (23)	12 (16)	
IIA	13 (9)	12 (16)	
IIB	30 (22)	21 (28)	
IIIA	8 (6)	2 (3)	
IIIB	36 (26)	17 (23)	
IVA	4 (3)	0 (0)	
IVB	5 (4)	1 (1)	
Uterine cervix tumor pathology			
Mean tumor diameter ± SE (range), centimeter	5.0 ± 0.1 (0.9–10.0)	4.7 ± 0.2 (0.7–7.2)	0.11
Deep third stromal invasion	128 (93)	65 (88)	0.23
Histologic Grade 3	88 (64)	44 (59)	0.54
Angiolymphatic invasion, positive	66 (48)	33 (46)	0.65
Lymph node metastases			
Pelvic nodal metastases	60 (43)	30 (41)	0.68
Para-aortic nodal metastases	22 (16)	12 (16)	0.74
Anemia (hemoglobin < 10 mg/dl)	50 (36)	26 (35)	0.87
Renal insufficiency (creatinine > 1.5 mg/dl)	12 (9)	3 (4)	0.21
Surgery			
Hysterectomy	33 (24)	22 (30)	0.38
Lymphadenectomy	25 (18)	14 (19)	0.88
Radiotherapy			
Median external-beam dose, cGy	4,500	4,500	0.97
Median LDR brachytherapy dose, cGy	4,000	4,000	0.12
Median HDR brachytherapy dose, cGy	2,500	2,500	0.56
Median treatment course, days (RDI)	68 (0.82)	56 (1.00)	<0.001
Chemotherapy			
Indication for concurrent chemotherapy	117 (85)	64 (86)	0.74
Five or six cycles of cisplatin 40 mg m^−2^	75 (64)	41 (64)	0.99
			

*Because of rounding, not all percentages total 100. AppKY, Appalachian Kentucky residence; cGy, centigray; RDI, relative dose intensity; SE, standard error of the mean.

### Statistical Analyses

Pathological findings were scored according to the classifications for manual tumor diameter, fractional depth of invasion of tumor into cervical stroma, tumor grade, and angiolymphatic space invasion as described before (4). For this study, women were considered to have received definitive external-beam radiotherapy plus chemotherapy if there were three or more infusions of chemotherapy during the dates of any radiotherapy (14). Relative radiotherapy dose (RD) was defined as the ratio of the actual administered dose of radiotherapy to the expected administered dose of radiotherapy (i.e., LDR 8,000 cGy or HDR 7,000 cGy). Relative time (RT) was defined as the ratio of the actual duration to the expected duration of radiotherapy (i.e., 8 weeks). Relative dose intensity (RDI) represented the ratio of relative dose to relative time (RD/RT). A ratio less than 1 indicated that women received less intense radiotherapy than planned (14). Full treatment was defined as completing within 8 weeks of radiotherapy because this was the proposed treatment span in clinical trials (8–13). First cancer relapse was scored as a central pelvic recurrence if it was documented within the radiation field and scored distant if elsewhere. Pearson’s chi-square test for categorical variables or Student’s *t-*test for continuous variables was computed. The Kaplan–Meier method was used to calculate a survival estimate, and differences in survival by AppKY residence were evaluated by the log-rank test. A *p-*value *α* of less than 0.05 (two-sided) determined statistical significance. Statistical software was used (Microsoft Corporation, 2019. Microsoft Excel. Available at: https://office.microsoft.com/excel).

## Results

### Characteristics of Patients

A total of 212 (25%) of 852 women treated at UKY between January 2001 and July 2021 were reviewed; 138 (65%) resided in AppKY and 74 (35%) lived elsewhere in central or eastern Kentucky. The characteristics of the two AppKY and non-AppKY cohorts are summarized in **Table 1**. Reflective of the region, more Hispanic or Latino women resided in urban manufacturing non-AppKY counties than rural agricultural AppKY counties (*p* = 0.02). Age at diagnosis was not different between the cohorts (*p* = 0.41). The median age at diagnosis was 54 years in our combined cohort, which is older than the median 50 years across the US (1). In AppKY, UCCx was most frequently diagnosed among women aged 55–64 years (**Figure 1**), which is up to two decades older than the US national trend [35–44 years, ref. (1)]. There were no differences in UCCx pathological characteristics between the cohorts (**Table 1**). Stage at diagnosis was not different between the AppKY and non-AppKY cohorts (*p* = 0.12)—overall, 29% were localized (confined to the primary site), 52% were regional (spread beyond the primary site or spread to regional pelvic lymph nodes), and 19% were distant (metastasized). In AppKY, UCCx stage at diagnosis was 30% localized, 50% regional, and 20% distant. For context, US national averages for UCCx stage at diagnosis are 44% localized, 36% regional, and 16% distant (1). In 82 women with UCCx squamous cell cancers without paraaortic node metastases from AppKY, 88% of women had tumors estimated at 3 cm or more in diameter, 93% had deep third invasion, 56% had grade 3 histology, and 39% had angiolymphatic invasion. In 37 women with UCCx squamous cell cancers without paraaortic node metastases from non-AppKY, 84% of women had tumors estimated at three centimeters or more in diameter, 84% had deep third invasion, 60% had grade 3 histology, and 38% had angiolymphatic invasion. For these squamous cell cancers, tumor diameter (*p* = 0.55), deep third invasion (*p* = 0.21), histologic grade (*p* = 0.65), and presence of angiolymphatic invasion (*p* = 0.90) were not different among the AppKY and non-AppKY cohorts.

### Treatment and Compliance

Radiotherapy was delivered according to prescription or with minor deviations in 93% among those from AppKY and 92% in those from non-AppKY. Major but acceptable deviations occurred in another 4% and 7% of these cohorts, respectively. Four patients (2%) did not undergo planned brachytherapy—two patients refused and two patients did not undergo it for other reasons. In all, 26 (12%) patients did not complete prescribed radiotherapy. Of the 138 AppKY patients who had indications for radiotherapy, 111 (80%) patients sought external-beam radiotherapy closer to their home residence. If done, brachytherapy was conducted at UKY (98%). The median external-beam, LDR, and HDR RD intensity were not different among cohorts (**Table 1**). The median total duration of external-beam radiotherapy plus brachytherapy was 68 days in the AppKY cohort and 56 days in the non-AppKY cohort, for a significantly lower RT intensity in the AppKY cohort (*p* < 0.001). The RDI was 0.82 in the AppKY cohort and was 1.00 in the non-AppKY cohort (*p* < 0.001).

Of the 181 patients who had indications for chemotherapy during radiotherapy, 177 (98%) patients completed at least three courses of chemotherapy, and 116 (64%) patients completed five or six weekly cycles. There were no significant differences in chemotherapy administration between AppKY and non-AppKY cohorts (*p* = 0.96). Chemotherapy was discontinued because of toxic effects in 23 patients, refusal to continue in seven patients, diminished performance status in 11 patients, and other reasons in 24 patients.

Of the 55 patients who underwent radical surgery, 19 (35%) patients did so after radiotherapy for an intent to reduce UCCx central pelvic relapse. Of the 19 patients, nine (47%) patients underwent lymphadenectomy.

### Outcome

The median follow-up was 26 months for AppKY and 30 months for non-AppKY cohorts (*p* = 0.11). Follow-up data were available for all 212 patients. Of these, 51 (37%) AppKY patients and 24 (32%) non-AppKY patients were alive with no evidence of disease (*p* = 0.51). In addition, 14 (10%) AppKY and eight (11%) non-AppKY patients were alive but had recurrent UCCx (*p* = 0.88). The rate of first central pelvic relapse was 16% in the AppKY cohort and 9% in the non-AppKY cohort (*p* = 0.19). The rate of first distant relapse was 20% in the AppKY cohort and 28% in the non-AppKY cohort (*p* = 0.18). Overall, a 5-year survival estimate for UCCx is 59% (95% confidence interval: 51%–66%), whereas the US national 5-year estimate is 66% for this disease (1). The 5-year survival estimates were 58% (95% confidence interval: 47%–67%) in the AppKY and 61% (95% confidence interval: 48%–73%) in the non-AppKY cohorts, respectively (*p* = 0.43).

## Discussion

We found that in the UKY catchment, the majority of women with UCCx resided in AppKY and that their stage at diagnosis was more advanced than observed in the US general population. Most women from AppKY sought their external-beam radiotherapy in AppKY and then returned to UKY for brachytherapy. The radiotherapy course was up to 2 weeks longer in women from AppKY, resulting in a lower intensity of treatment and in a higher rate of central pelvic relapse. Although patients having indications for chemotherapy during radiotherapy actually started treatment, 36% of patients did not receive five or six weekly cisplatin cycles as planned for their course. In all, we observed a 7% lower 5-year survival estimate for women with UCCx living in the entire UKY catchment as compared to UCCx survivors from the US general population.

Appalachian poverty, unemployment, and lower levels of education are major socioeconomic problems, with 25% of women in the region impoverished as compared to the 15% rate across the US (15). In AppKY, women have insufficient access to and knowledge of cancer screening programs (15), likely major drivers of the extraordinary UCCx healthcare burden in the region. It is noteworthy that our finding of higher percentages of advanced-stage UCCx at diagnosis tracks closely with Appalachian poverty indicators found in eastern Kentucky. Moreover, the elevated rates of poverty and lower levels of education also implicate smoking as a contributor to the increased incidence and poor prognosis of UCCx (16–18). Smoking in women confers nearly double the risk of invasive UCCx and predicts worse overall survival in women with advanced-stage UCCx treated by radiotherapy plus chemotherapy (17, 18). It again is remarkable that in our AppKY cohort, 64% of women were current smokers and had more advanced-stage UCCx disease at diagnosis than expected for the US UCCx population. The negative effects of poverty, lower levels of education, and smoking on UCCx radiotherapy plus chemotherapy treatment compliance deserve further research, especially if these factors are obstacles to US National Cancer Institute clinical trial participation.

The interaction between socioeconomic factors, smoking, and age is complex for UCCx when considered for Appalachia because the use of the Papanicolaou (Pap) exfoliative cytology screening test for detection of precancerous lesions has a decreasing gradient with age (17). In our study, UCCx in Kentucky was most frequently diagnosed among women aged 55–64 years, with a median of 54 years, which is comparable with the US national trend of 50 years (1). In our cohort, Pap test results with reflex human papillomavirus (HPV) testing were not reliably abstracted due to incomplete non-oncology medical records. The observation of an older UCCx patient population in the UKY catchment may be explained by variable public health intervention practices, and possibly, Appalachian poverty and lower levels of education, but further research is needed.

Prior Gynecologic Oncology Group studies of external-beam radiotherapy followed by brachytherapy have used a 56-day course of treatment as the benchmark for sufficient relative RT intensity (8–13). This benchmark has been based on observations that central pelvic control decreased 0.7% per day and survival decreased 0.6% per day for each additional day of treatment beyond 55 days for all stages of disease (19, 20). In our cohorts, women residing in AppKY had a median 68-day course of treatment for an RDI of 0.82, meaning these women had a lower intensity of cancer treatment. Our treatment compliance findings indicate that local radiotherapy plus chemotherapy delivery was generally on time, but an in-time window return to UKY for brachytherapy was delayed consistently. Our research was unable to abstract reasons for the delay in brachytherapy, but it is not unreasonable to suggest that Appalachian poverty and lack of reliable transportation are contributors to the delay (15).

A prospective surgical pathological Gynecologic Oncology Group study of 732 subjects with presurgical stage I UCCx squamous cell cancers (representing 78% of the 940 evaluable subjects) found a 16% rate of pelvic node metastasis and a 4% rate of paraaortic node metastasis (4). The study mandated subjects to have more than 3 mm of invasion, but did not prespecify stage IB disease at diagnosis. Women with adenocarcinoma (12%) or adenosquamous cancers (8%) were not further reported on in the manuscript. In 645 subjects with UCCx squamous cell cancers without paraaortic node metastases, 24% of subjects had tumors estimated at 3 cm or more in diameter, 36% had deep third invasion, 28% had grade 3 histology, and 43% had angiolymphatic invasion. Our study of 129 women with any-stage UCCx squamous cell cancers (representing 66% of the 212 evaluable patients) observed a 47% rate of pelvic node metastasis and a 16% rate of paraaortic node metastasis. In 119 women with UCCx squamous cell cancers without paraaortic node metastases, 87% of women had tumors estimated at 3 cm or more in diameter, 90% had deep third invasion, 57% had grade 3 histology, and 39% had angiolymphatic invasion. Together, our findings suggest a pathologically more aggressive pattern of disease in the Appalachian and bordering regions of Kentucky. Such observations may be explained again by variable public health intervention practices, and possibly, Appalachian poverty and lower levels of education. However, it is also important to take into account that estimating tumor diameter by palpation is subjective, imprecise, and cannot be verified for quality control. Assessment of histologic grade and presence or absence of angiolymphatic invasion is also subjective due to variability in tumor sampling and discrepancy in microscopic review.

Additional data on relapse and survival after further follow-up and molecular case review might provide a clearer assessment of Appalachian women with UCCx seeking radiochemotherapy who require additional adjuvant or alternate treatment.

## Data Availability Statement

The raw data supporting the conclusions of this article will be made available by the authors, without undue reservation.

## Ethics Statement

The studies involving human participants were reviewed and approved by the University of Kentucky. Written informed consent for participation was not required for this study in accordance with the national legislation and the institutional requirements.

## Author Contributions

CK, DF, MK, RM, FU, and MR contributed to the collection and review of any data, analysis, authentication, and the writing and approval of this manuscript. The views expressed are those of the authors and not those of the University of Kentucky. Links or discussions of specific treatments do not constitute endorsement. All authors contributed to the article and approved the submitted version.

## Funding

This work was supported by NCI grant P30 CA177558, which supports the Biostatistics and Bioinformatics and the Cancer Research Informatics Shared Resource Facilities of the University of Kentucky Markey Cancer Center.

## Conflict of Interest

The authors declare that the research was conducted in the absence of any commercial or financial relationships that could be construed as a potential conflict of interest.

## Publisher’s Note

All claims expressed in this article are solely those of the authors and do not necessarily represent those of their affiliated organizations, or those of the publisher, the editors and the reviewers. Any product that may be evaluated in this article, or claim that may be made by its manufacturer, is not guaranteed or endorsed by the publisher.
